# Clinical practice in the management of enteroatmospheric fistula and fistuloclysis: a case report

**DOI:** 10.1590/1980-220X-REEUSP-2024-0369en

**Published:** 2025-02-07

**Authors:** Glorinha Pereira Alves, Larissa Carvalho de Castro, Juliano Teixeira Moraes, Daniel Nogueira Cortez

**Affiliations:** 1 Faculdade Ciências Médicas, Belo Horizonte, MG, Brazil.; 2 Universidade Federal de São João del Rei, Divinópolis, MG, Brazil.

**Keywords:** Jejunostomy, Intestinal Fistula, Nutrition Therapy, Enterostomal Therapy, Yeyunostomía, Fístula Intestinal, Terapia Nutricional, Estomaterapia

## Abstract

**Objective:**

To describe the intervention of fistuloclysis in enteroatmospheric fistulas.

**Method:**

This is a descriptive case report constructed according to the Consensus-based Clinical Case Reporting Guideline Development, carried out with a male patient in a medium-sized philanthropic hospital in a city in Minas Gerais, Brazil. The study was previously approved by a Research Ethics Committee.

**Results:**

T: Patient with a history of radical prostatectomy and elective cholecystectomy, with progression of colon ischemic necrosis, which required left colectomy and Hartmann colostomy, developed spontaneous enteroatmospheric fistulas and was on enteral nutrition for nine months. Due to complications, fistuloclysis was initiated, which resulted in significant nutritional improvement.

**Conclusion:**

The description of the procedure and care allows its reproduction in a safe manner for effluent control, nutritional restoration, and other clinical responses. The care provided by the stomatherapy nurse stands out, taking into account the benefits, complexity, and challenges of fistuloclysis.

## RESUMEN

**Objetivo:**

Describir la intervención de fistuloclisis en fístulas enteroatmosféricas.

**Método:**

Se trata de un reporte de caso descriptivo construido según *Consensus-based Clinical Case Reporting Guideline Development*, realizado con un paciente masculino en un hospital filantrópico de tamaño mediano en una ciudad de Minas Gerais, Brasil. El estudio fue previamente aprobado por un Comité de Ética e Investigación.

**Resultados:**

Paciente con antecedentes de prostatectomía radical y colecistectomía electiva, con evolución de necrosis isquémica del colon, que requirió colectomía izquierda y colostomía de *Hartman*, desarrolló fístulas enteroatmosféricas espontáneas y estuvo en nutrición enteral durante nueve meses. Por complicaciones se inició fistuloclisis, lo que resultó en importante mejoría nutricional.

**Conclusión:**

La descripción del procedimiento y cuidados permite su reproducción segura para el control de efluentes, restauración nutricional y otras respuestas clínicas. Se destaca la atención brindada por el enfermero estomatólogo, teniendo en cuenta los beneficios, complejidad y desafíos de la fistuloclisis.

## INTRODUCTION

Enteroatmospheric fistulas (EAF) are abnormal communications between the intestine and the abdominal skin^(1,2,3)^. EAFs can arise from intestinal perforations resulting from complications of abdominal trauma, inflammatory bowel diseases, peptic ulcers, diverticulitis, abdominal surgical procedures, presence of foreign bodies in the intestine, among other causes^(1,4,5)^.

EAFs can be classified, according to location, into proximal (stomach, duodenum, jejunum, or proximal ileum) and distal (distal ileum or colon); according to the amount of effluent, into low (<200 ml/24h), moderate (200 to 500 ml/24h), and high (>500 ml/24h); as to the location in the abdomen, in superficial (drains the intestinal contents through the wound in the abdominal cavity) and deep (drains into the abdominal cavity); and as to the number of fistulas, in single, multiple fistulas in close proximity (two or more fistulas close to each other) and multiple distant fistulas (two or more fistulas distant from each other)^(6,7)^.

Fluid and electrolyte loss and acid-base imbalance increase morbidity rates, with potential for mortality^(8)^. Mortality varies between 6% and 33% and the incidence varies depending on the etiology, with pancreatic infection having a high rate of 50%, trauma between 2% and 25%, and abdominal sepsis between 20% and 25%^(9,10)^.

EAF presents a challenge for clinical practice due to its complexity associated with the patient’s critical conditions^(11)^. The output generated by the EAF is directly related to the patient’s critical evolution. The care of patients with EAF involves, among others, psychological support, attention to dermatological care protocols, implementation of strict measures against sepsis and management of fistuloclysis.

Fistuloclysis is the reinfusion of effluent from a proximal fistula, through a catheter, into a distal segment^(4,12)^. It provides correction of hydroelectrolytic imbalances and effective management of fistula flow measurement, to improve clinical, nutritional and immunological conditions, increasing the chances of success in future fistula reconstruction procedures^(13)^

**Figure 1:**
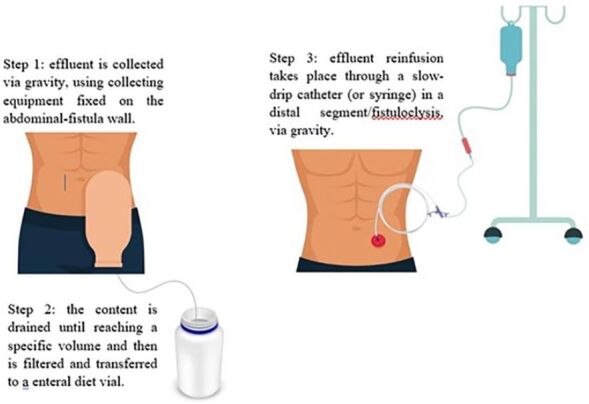
Technique used to perform fistuloclysis.

The technique for performing fistuloclysis uses the distal segment of the fistula itself as a route of nutritional infusion, which may include enteral nutrition or reinfusion of effluent from the proximal fistula to reabsorb nutrients (Figure 1)^(1)^.

Although the fistulolysis technique is little explored in Brazilian literature, the procedure is an alternative means of nutritional support under the guidance of a stomatherapy nurse, offering advantages as it is safe, reliable, and of low cost^(12)^. The importance of the stomatherapist specialist in carrying out the procedure is highlighted, given their training. In this regard, the objective of this study was to describe the intervention of fistuloclysis in enteroatmospheric fistulas.

## METHOD

### Design of Study

This is a descriptive case report, prepared in accordance with the CARE guidelines (*CASe REports*) developed by an international group of experts to support an increase in the accuracy, transparency, and usefulness of case reports^(14)^.

### Data Collection

Data were collected through clinical evaluation and records made in a patient’s hospital record, from February 19 to March 29, 2024 in a medium-sized philanthropic hospital located in a municipality in Minas Gerais, Brazil.

### Ethical Aspects

The case study was authorized by the patient and a free and informed consent form was signed as approved by an Ethics Committee with opinion no. 6.651.501.

## RESULTS

Male patient, 76 years old, diagnosed with Systemic Arterial Hypertension (SAH) and Chronic Obstructive Pulmonary Disease (COPD). Smoker for 50 years, with a surgical history of radical prostatectomy because of prostate cancer.

On 10/26/2022, he underwent cholecystectomy due to cholelithiasis and cholecystitis, and was discharged from the hospital on 10/27/22 in the morning. On the same day, the patient returned to the hospital with abdominal pain and bladder swelling due to urethral stenosis. He underwent emergency cystostomy, progressing with clinical worsening, abdominal pain and distension, persistent vomiting, and ineffective respiratory pattern related to paralytic ileus.

**Figure 2:**
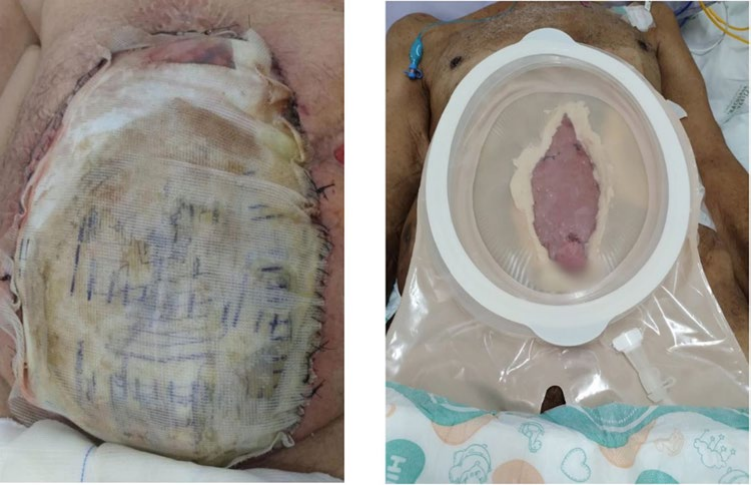
Implanted bogota bag and large fistula system.

After evaluating the results of the abdominal computed tomography scan, the presence of free fluid in the peritoneal cavity was observed. Subsequently, an abdominal paracentesis was performed, revealing the presence of fluid with biochemical characteristics consistent with bile. Undergoing laparotomy, choleperitoneum with ischemic necrosis of the left colon was found, resulting in the need for a left colectomy, followed by the creation of a Hartmann colostomy, cleaning of the peritoneal cavity, treatment of cholestasis of the ducts of Luschka and alternative closure by laparotomy with a Bogota bag (Figure 2). The patient progressed with spontaneous EAF formation on the laparotomy plate on 11/12/2022. EAF presented with wide, labiate, everted fistulae, with high output of effluent secretion around 500 to 800 ml per day collected by a large fistula system (Figure 2).

Afterwards, the patient remained hospitalized for nine months in the intensive care unit, alternating with the ward unit, during which time the patient presented clinical and surgical complications. The main complications recorded were: pneumonia, urinary tract infection, tracheitis, peritonitis, recurrent severe septic shock, mesenteric vascular disease, stage IV pressure injury in the sacral and trochanteric region, shingles, renal dysfunction, surgical wound infection, irritant contact dermatitis, malnutrition, oropharyngeal dysphagia, immobility syndrome, psychic depression, anemia, polyneuromyopathy, central venous access infection, thrombophlebitis and venous thrombosis of the left internal and right external jugular veins, soft tissue cellulitis of the cervical region. During the hospitalization period, the patient remained on enteral and parenteral nutrition. These complications prevented the continuation of the parenteral route for nutrition on 06/26/2023, with enteral nutrition remaining.

**Figure 3:**
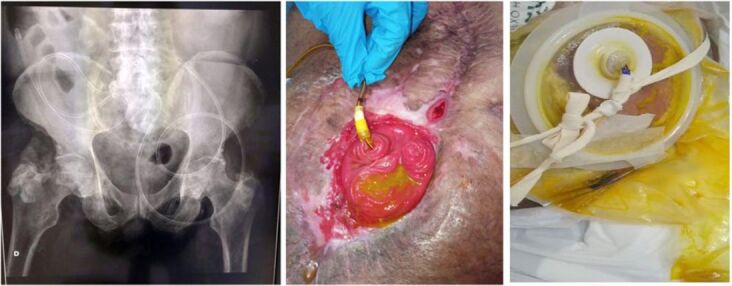
Image of the endoscopic route for passing a catheter into the jejunostomy.

On 06/30/2023, as enteral nutrition was not sufficient to maintain clinical and nutritional parameters, it was decided that fistuloclysis would be implemented. The patient underwent passage of nasoenteric catheter endoscopically in the distal loop of the fistula as part of the treatment, establishing a jejunostomy (Figure 3). Due to the lack of information in the literature on catheter fixation, it was adapted to a height demarcation and the catheter was fixed with blue adhesive tape in the image and a cord passed through the abdominal region, to ensure the maintenance of the correct position, avoiding its translocation. The white sticker around the catheter and attached to the collection device is a button that helps hold the catheter in place (Figure 3).

**Figure 4:**
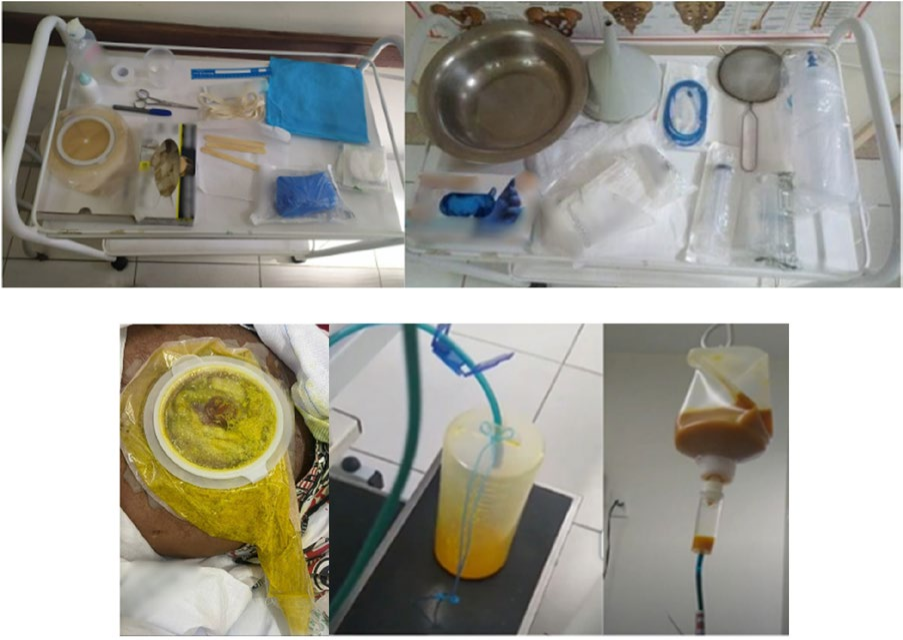
Equipment used for the fistuloclysis procedure and removal of effluent from the collection equipment, open system for collection and reinfusion through an infusion set after filtration.

The materials required for the fistuloclysis technique were: size 12 nasoenteric catheter, one-piece collection equipment with a 100 mm inspection window indicated for postoperative use, open system for effluent collection, 60 ml diet vial or syringe, nutrition equipment, filter for separating larger particles from the contents and collection bottle (Figure 4).

Effluent collection was performed via gravity, using a colostomy collection device with a 100 mm window attached to the abdominal fistula wall. The contents were drained until reaching a volume of 200 ml and then filtered and transferred to an enteral diet vial. The effluent was infused through a slow-drip catheter implanted for fistuloclysis, via gravity, using a macrodropper device (Figure 4). When the viscosity was higher, manual fistuloclysis was required, using a 60 ml syringe in slow infusion. The procedure was performed up to twice a day depending on the drained volume reaching 200 ml.

After starting fistuloclysis, an improvement in hydration, nutritional status, and anthropometric measurements was observed (Table 1).

**Table 1:** Presentation of the evolution of clinical variables before and after fistuloclysis - Curvelo, MG, Brazil, 2024.

Clinical variables	06/27/2023	07/16/2023
Hemoglobin	9.50 g/dl	9.80 g/dl
Hematocrit	28.50%	29.60%
Platelets	119.0 thousand/mm^3^	201.0 thousand/mm^3^
Total proteins	5.1 g/dl	5.4 g/dl
Albumin	2.4 g/dl	2.6 g/dl
Globulin	2.7 g/dl	2.8 g/dl
Weight	60 kg	68.8 kg
Arm circumference	24 cm	30 cm
Calf circumference	26 cm	28 cm

Family members were accompanied in the care provided to the patient in the last days of hospitalization as a way of training and preparing for discharge.

For the family to continue the procedure at home, the following guidelines were provided: change and adaption of the collection equipment; cutting of the equipment according to the diameter of the fistula; use of adjuvants such as powder and paste for ostomy; skin care; management of effluent collection; care with reinfusion, taking into account the need to pass the effluent through the filter; management of fistuloclysis; sanitation of all materials used; importance of paying attention to the marking of the catheter to avoid translocation.

The patient was discharged on 07/18/2023 after verifying that the family members were able to place the collection system in the abdominal region and perform the fistuloclysis procedure safely.

Treatment continued at home under the supervision of the hospital’s reference stomatherapy nurse with family participation. A new surgical intervention was planned to resect the fistulous segment and reconstruct the intestinal transit, scheduled 6 to 12 months after hospital discharge.

## DISCUSSION

The procedure established the basis for the patient’s nutritional and physiological recovery, allowing for overall clinical improvement. Technical skill, combined with creativity, allows nurses to create strategies to protect the skin and restrain effluent, even in adverse conditions. The process of evaluating the nursing team’s learning in relation to the provision of care, patient monitoring, and recording of actions constitute determining factors for nursing care quality and safety^(3)^.

EAF management poses a significant challenge to enterostomal nurses. These include peri-fistula skin care, effluent measurement, proper use of collection devices, and guarantee of nutrition through the fistuloclysis technique. Reinfusion of effluent into the catheter allows nutrients to be absorbed, using the distal segment of the fistula as a route for nutritional administration^(1)^.

Enteral nutrition through fistuloclysis is an effective alternative for some patients with secondary intestinal fistula in open abdomen, which should not replace parenteral nutrition in all cases, but may help reduce hospital stay^(1)^.

Fistuloclysis is a procedure recognized for its safety and effectiveness. Proper management of effluent in high-output fistulas is essential to ensure the benefits of enteral nutrition when applied in situations where the distal fistula serves as a gateway for the administration of enteral formulas or gastrointestinal secretions^(1,15,16)^.

Depending on the fistula location, the effluent may contain significant amounts of salivary amylase, gastric pepsin, pancreatic enzymes and bile that allow the reestablishment of circulation and enterohepatic function and benefit the patient’s recovery^(1)^.

Several studies have confirmed the safety and reduction of morbidity and mortality in patients who underwent fistuloclysis, without reporting adverse effects directly attributable to enteral nutrition^(1,15,17,18,19)^.

In one of the studies, 95 patients were analyzed, of which 35 used fistuloclysis with reinfusion of enteric juice, observing satisfactory tolerance, improvement in liver function, and reduction in distal output^(15)^. Another study mentioned the increased survival of all patients undergoing this nutritional method^(18)^. In a Czech study, it was demonstrated that fistuloclysis improved liver function more than enteral nutrition, leading to faster optimization of nutritional parameters, reducing hospital stay and reducing overall mortality of cases^(19)^.

There are several implementation methods, among which we can highlight the one used in this study. Methods range from application through gravity to sophisticated closed systems with the possibility of continuous applications^(20,21)^.

The most common risk of the procedure is the displacement of the catheter, which can occur to the outside due to incorrect handling or obstruction, or internalization due to intestinal peristalsis^(19)^.

Fistuloclysis is successful when the team, caregivers, and/or family members are involved in the process^(22)^. In this study, bedside training, conducted among the nursing team and family members, faced resistance, initially due to a lack of understanding about the importance of the procedure. However, this resistance was reduced after training and detailed explanations about the importance of the method. For those who were unable to attend the in-person training, an instructional video was created, with a step-by-step guide to the procedure. To ensure a safe discharge, family members care was monitored during the last days of hospitalization to resolve any doubts.

Fistuloclysis management presented significant barriers from stomatherapy nurses and patients, with the refusal to administer the enteric content that was removed from the organism itself and aesthetic concerns on the part of patients and family members. Constant and adequate education by the multidisciplinary team can reduce these barriers^(1,2,23)^.

Collaboration among the patient, family, and team will facilitate a comprehensive and effective approach to care. Each team member should conduct assessments specific to their expertise and collaborate with other team members before establishing mutually agreed upon goals. Goals should include maintaining comfort and dignity, meeting the patient’s psychosocial needs, and promoting their independence^(24)^.

The work of the specialist stomatherapist nurse stands out, as they play a fundamental role in healthcare, especially in the care of people with stomas and EAF. This professional is highly specialized and trained to provide technical and emotional support to improve patients’ quality of life^(25)^.

As implications and potential for practice, it is possible to state that fistuloclysis provides a nutritional response that improves the patient’s clinical condition and recovery. The improvement in the condition provided by the procedure has a positive impact on quality of life and speeds up hospital discharge. Fistuloclysis can be managed at home, as long as family members are trained and supervised.

Limitations should be considered as the lack of pre-established protocols for performing and caring for fistuloclysis. The references used are publications from other countries that may differ due to access to technologies and other resources not available in Brazil. Another limitation is the fact that this is a single case to be described in the study, but refers to the absence of other patients who underwent the same procedure. It is also important to highlight the difficulty and resistance by both health professionals and family members in carrying out the procedure.

The description of the fistuloclysis technique associated with discussions about the case can provide support for other professionals in Brazil to develop the procedure and publish new cases in journals and scientific events.

## CONCLUSION

The description of the procedure and care allows its reproduction in a safe manner for effluent control, nutritional restoration, and other clinical responses. The care provided by the stomatherapy nurse stands out, taking into account the benefits, complexity, and challenges of fistuloclysis.
