# Supplementary material for: Clinical practice in the management of enteroatmospheric fistula and fistuloclysis: a case report
Source: Rev Esc Enferm USP. 2025 Feb 7;58:e20240369. doi: 10.1590/1980-220X-REEUSP-2024-0369en (PMC11835223; doi:10.1590/1980-220X-REEUSP-2024-0369en)
Supplement: Translation

# Translation: Prática clínica no manejo da fístula enteroatmosférica e fistuloclise: relato de caso

**DOI:** 10.1590/1980-220X-REEUSP-2024-0369pt

**Authors:** Glorinha Pereira Alves, Larissa Carvalho de Castro, Juliano Teixeira Moraes, Daniel Nogueira Cortez

**Affiliations:** 1 Faculdade Ciências Médicas, Belo Horizonte, MG, Brasil.; 2 Universidade Federal de São João del Rei, Divinópolis, MG, Brasil.

**Keywords:** Jejunostomia, Fístula Intestinal, Terapia Nutricional, Estomaterapia

## Abstract

**Objetivo:**

Descrever a intervenção de fistuloclise em fístulas enteroatmosféricas.

**Método:**

Trata-se de um relato de caso descritivo construído conforme *Consensus-based Clinical Case Reporting Guideline Development*, realizado com paciente do sexo masculino em um hospital filantrópico de médio porte de um município de Minas Gerais, Brasil. O estudo foi aprovado previamente por um Comitê de Ética e Pesquisa.

**Resultados:**

Paciente com histórico de prostatectomia radical e colecistectomia eletiva, com evolução de necrose isquêmica do cólon, que necessitou de colectomia esquerda e colostomia de *Hartmann, d*esenvolveu fístulas enteroatmosféricas espontâneas e estava em nutrição enteral por nove meses. Devido a complicações, iniciou a fistuloclise, que resultou em melhora nutricional significativa.

**Conclusão:**

A descrição do procedimento e dos cuidados permite sua reprodução de forma segura para o controle de efluentes, restauração nutricional e demais respostas clínicas. Destacam-se os cuidados prestados pelo enfermeiro estomaterapeuta, levando em conta os benefícios, a complexidade e os desafios da fistuloclise.

## INTRODUÇÃO

Fístulas enteroatmosféricas (FEA) são comunicações anormais entre o intestino e a pele abdominal^(1,2,3)^. As FEA podem surgir por perfurações intestinais provenientes de complicações por trauma abdominal, doenças inflamatórias intestinais, úlceras pépticas, diverticulite, procedimentos cirúrgicos abdominais, presença de corpos estranhos no intestino, entre outras causas^(1,4,5)^.

As FEA podem ser classificadas, quanto à localização, em proximal (estômago, duodeno, jejuno ou íleo proximal) e distal (íleo distal ou cólon); quanto à quantidade de efluente, em baixa (<200 ml/24h), moderada (200 a 500 ml/24h) e alta (>500 ml/24h); quanto à localização no abdome, em superficial (drena o conteúdo intestinal através da ferida na cavidade abdominal) e profunda (drena para dentro da cavidade abdominal); e quanto ao número de fístulas, em única, múltiplas fístulas próximas (duas ou mais fístulas próximas umas das outras) e múltiplas fístulas distantes (duas ou mais fístulas distantes umas das outras^(6,7)^.

A perda de fluidos e eletrólitos e desequilíbrio ácido-base aumentam as taxas de morbidade, com potencial para mortalidade^(8)^. A mortalidade varia entre 6% a 33% e a incidência é variável conforme a etiologia, sendo a infecção pancreática com alta taxa de 50%, trauma entre 2% e 25% e sepse abdominal entre 20% e 25%^(9,10)^.

A FEA apresenta-se como um desafio para a prática clínica pela sua complexidade associada às condições críticas do paciente^(11)^. A quantidade de débito gerada pela FEA tem relação direta com a evolução crítica do paciente. O cuidado do paciente com FEA envolve, entre outros, suporte psicológico, atenção aos protocolos de cuidados dermatológicos, implementação de medidas rigorosas contra a sepse e o manejo da fistuloclise.

A fistuloclise é a reinfusão do efluente de uma fístula proximal, por meio de um cateter, para um segmento distal^(4,12)^. Ela proporciona a correção dos desequilíbrios hidroeletrolíticos e a gestão eficaz da mensuração do fluxo da fístula, para melhorar as condições clínicas, nutricionais e imunológicas, aumentando as chances de sucesso no procedimento futuro para reconstrução da fístula^(13)^

A técnica para a realização da fistuloclise utiliza o próprio segmento distal da fístula como via de infusão nutricional, que pode incluir a nutrição enteral ou a reinfusão do efluente proveniente da fístula proximal com o objetivo de reabsorção dos nutrientes (Figura 1)^(1)^.

**Figura 1:** Técnica utilizada para a realização da fistuloclise.

Embora a técnica de ﬁstuloclise seja pouco explorada na literatura brasileira, o procedimento é um meio alternativo de suporte nutricional sob a condução do enfermeiro estomaterapeuta, que oferece vantagens por ser seguro, confiável e de baixo custo^(12)^. Destaca-se a importância do especialista estomaterapeuta na realização do procedimento diante de sua formação. Neste sentido, o objetivo deste estudo foi descrever a intervenção de fistuloclise em fístulas enteroatmosféricas.

## MÉTODO

### Desenho do Estudo

Trata-se de um relato de caso descritivo, elaborado em conformidade com as diretrizes CARE (*CAse REports*) desenvolvidas por um grupo internacional de especialistas para apoiar um aumento na precisão, transparência e utilidade dos relatórios de casos^(14)^.

### Coleta de Dados

Os dados foram coletados por meio da avaliação clínica e dos registros feitos no prontuário hospitalar de um paciente, durante o período de 19 de fevereiro a 29 de março de 2024 em um hospital filantrópico de médio porte situado em um município de Minas Gerais, Brasil.

### Aspectos Éticos

O estudo do caso foi autorizado pelo paciente e um termo de consentimento livre e esclarecido foi assinado conforme aprovação por um Comitê de Ética sob o parecer nº. 6.651.501.

## RESULTADOS

Paciente, sexo masculino, 76 anos, com diagnóstico de Hipertensão Arterial Sistêmica (HAS) e Doença Pulmonar Obstrutiva Crônica (DPOC). Tabagista há 50 anos, com passado cirúrgico de prostatectomia radical devido a um câncer de próstata.

No dia 26/10/2022 foi submetido à colecistectomia em decorrência de colelitíase e colecistite, com alta hospitalar em 27/10/22 pela manhã. No mesmo dia, o paciente retornou ao hospital apresentando dor abdominal e bexigoma, devido à estenose de uretra. Foi submetido à cistostomia de urgência evoluindo com piora clínica, dor e distensão abdominal, vômitos persistentes e padrão respiratório ineficaz relacionados ao íleo paralítico.

Após a avaliação do resultado da tomografia computadorizada do abdome, foi observada a presença de líquido livre na cavidade peritoneal. Subsequentemente, realizou-se uma paracentese abdominal, revelando a presença de líquido com características bioquímicas consistentes com bile. Submetido a laparotomia, foi identificado coleperitônio com necrose isquêmica do cólon esquerdo resultando na necessidade de uma colectomia esquerda, seguida pela criação de uma colostomia à *Hartmann*, higienização da cavidade peritoneal, tratamento da colestasia de *Luschka* e fechamento alternativo por laparotomia com bolsa de Bogotá (Figura 2). O paciente evoluiu com formação de FEA espontânea em placa de laparotomia em 12/11/2022. A FEA apresentou-se com fístulas amplas, labiadas, evertidas, com alto débito de secreção efluente em torno de 500 a 800 ml por dia coletados por sistema de fístula de tamanho grande (Figura 2).

**Figura 2:** Bolsa de Bogotá implantada e Sistema fístula de tamanho grande.

Após, o paciente submeteu-se a um período de internação hospitalar por nove meses no centro de tratamento intensivo, alternada com unidade de enfermaria, fase em que o paciente apresentou complicações clínicas e cirúrgicas. As principais complicações registradas foram: pneumonia, infecção de trato urinário, traqueítes, peritonites, choques sépticos graves recorrentes, doença vascular mesentérica, lesão por pressão em região sacral e trocantérica estágio lV, *Herpes Zoster*, disfunção renal, infecção de ferida operatória, dermatite irritativa de contato, desnutrição, disfagia orofaríngea, síndrome da imobilidade, depressão psíquica, anemia, polineuromiopatia, infecção de acesso venoso central, tromboflebite e trombose venosa das veias jugulares interna esquerda e externa direita, celulite de partes moles da região cervical. Durante o período de internação, o paciente permaneceu em nutrição enteral e parenteral. Estas complicações impediram a continuação da via parenteral para nutrição em 26/06/2023, permanecendo com a nutrição enteral.

Em 30/06/2023, em função da nutrição enteral não ser suficiente para manter os parâmetros clínicos e nutricionais, decidiu-se pela implantação da fistuloclise. O paciente foi submetido a passagem de cateter nasoentérico por via endoscópica na alça distal da fístula como parte do tratamento, estabelecendo uma jejunostomia (Figura 3). Devido à escassez de informações na literatura sobre a fixação do cateter, foi adaptada a uma demarcação de altura e fixado com fita adesiva de cor azul na imagem e cordão transpassado na região abdominal, com a finalidade de assegurar a manutenção da posição correta evitando sua translocação. O adesivo branco em volta do cateter e aderido ao equipamento coletor é um botom que ajuda a segurar o cateter (Figura 3).

**Figura 3:** Imagem da via endoscópica para passagem de cateter na jejunostomia.

Os materiais necessários para a técnica de fistuloclise foram: catéter nasoentérico tamanho 12, equipamento coletor do tipo de uma peça com janela de inspeção de 100 mm indicado para pós-operatório, sistema aberto para coleta do efluente, frasco de dieta ou seringa de 60 ml, equipo de nutrição, filtro para separação das partículas maiores do conteúdo e frasco coletor (Figura 4).

**Figura 4:** Equipamentos utilizados para o procedimento de fistuloclise e retirada do efluente do equipamento coletor, sistema aberto para coleta e reinfusão por equipo após filtragem.

A coleta do efluente foi realizada por via gravitacional, utilizando um equipamento coletor para colostomia com janela de 100 mm aderido à parede abdominal-fístula. O conteúdo era drenado até atingir um volume de 200 ml e, em seguida, filtrado por meio de um filtro e transferido para um frasco de dieta enteral. A infusão do efluente ocorria por meio de um cateter implantado para fistuloclise, de maneira gravitacional e com gotejamento lento, sendo utilizado um equipo de macrogotas (Figura 4). Quando a viscosidade estava maior, era necessário realizar a fistuloclise manual, com o uso de seringa de 60 ml em infusão lenta. O procedimento foi realizado até duas vezes ao dia conforme o volume drenado ao atingir 200 ml.

Após iniciar a fistuloclise, observou-se melhora na hidratação, estado nutricional e medidas antropométricas (Tabela 1).

**Tabela 1:** Apresentação da evolução das variáveis clínicas antes e após fistuloclise - Curvelo, MG, Brasil, 2024.

Variáveis clínicas	27/06/2023	16/07/2023
Hemoglobina	9,50 g/dl	9,80 g/dl
Hematócrito	28,50%	29,60%
Plaquetas	119,0 mil/mm^3^	201,0 mil/mm^3^
Proteínas totais	5,1 g/dl	5,4 g/dl
Albumina	2,4 g/dl	2,6 g/dl
Globulina	2,7 g/dl	2,8 g/dl
Peso	60 Kg	68,8 kg
Circunferência do braço	24 cm	30 cm
Circunferência da panturrilha	26 cm	28 cm

Os familiares foram acompanhados na realização dos cuidados prestados ao paciente nos últimos dias de internação como forma de capacitação e preparação para a alta.

Para a continuação do procedimento em domicílio pela família, foram realizadas as seguintes orientações: troca e adaptação do equipamento coletor; recorte do equipamento de acordo com diâmetro da fistula; uso de adjuvantes como pó e pasta para estomia; cuidados com a pele; manejo da coleta do efluente; cuidados com reifunsão, levando em consideração a necessidade de passar o efluente pelo filtro; manejo da fistuloclise; higienização de todos materiais de uso; importância de ficar atento à marcação do cateter para evitar translocação.

A alta foi realizada em 18/07/2023 após a verificação de que os familiares estavam aptos desde a colocação do sistema coletor na região abdominal e executavam o procedimento da fistuloclise com segurança.

O tratamento continuou em domicílio sob supervisão do enfermeiro estomaterapeuta de referência do hospital com participação da família. Foi planejada uma nova intervenção cirúrgica para ressecar o segmento fistuloso e reconstruir o trânsito intestinal, prevista de 6 a 12 meses após a alta hospitalar.

## DISCUSSÃO

O procedimento estabeleceu a base para a recuperação nutricional e fisiológica do paciente, permitindo a melhora clínica geral. A habilidade técnica, somada à criatividade, proporciona ao enfermeiro a criação de estratégias para a proteção da pele e contenção do efluente, mesmo em condições adversas. O processo de avaliação do aprendizado da equipe de enfermagem frente à prestação de cuidados, monitorização do paciente e registro das ações constituem fatores determinantes para a qualidade e a segurança na assistência de enfermagem^(3)^.

O manejo da FEA apresenta um desafio importante aos enfermeiros estomaterapeutas. Entre eles estão o cuidado da pele perifístula, medição do efluente, utilização adequada dos dispositivos coletores e garantia da nutrição por meio da técnica de fistuloclise. A reinfusão do efluente no cateter permite que os nutrientes sejam absorvidos, usando o segmento distal da fístula como via para a administração nutricional^(1)^.

A nutrição enteral por meio da fistuloclise é uma alternativa eficaz para alguns pacientes com fístula intestinal secundária em abdome aberto, que não deve substituir a nutrição parenteral em todos os casos, mas pode ajudar a reduzir o tempo de internação hospitalar^(1)^.

A fistuloclise é um procedimento reconhecido pela segurança e eficácia. A administração adequada do efluente em fístulas de alto débito é fundamental para assegurar os benefícios da nutrição enteral, quando aplicada em situações em que a fístula distal serve como porta de entrada para a administração de fórmulas enterais ou secreções gastrointestinais^(1,15,16)^.

De acordo com o local da fístula, o efluente pode conter quantidades significativas de amilase salivar, pepsina gástrica, enzimas pancreáticas e bile que permitem restabelecer a circulação e função entero-hepática e beneficiar a recuperação do paciente^(1)^.

Diversos estudos confirmaram a segurança e a redução da morbimortalidade em pacientes que receberam fistuloclise, sem relatar efeitos adversos atribuíveis diretamente à nutrição enteral^(1,15,17,18,19)^.

Em um dos estudos foram analisados 95 pacientes, dos quais 35 utilizaram a fistuloclise com reinfusão de suco entérico, observando uma tolerância satisfatória, melhoria na função hepática e redução do débito distal^(15)^. Já em outro foi mencionado o aumento da sobrevida de todos os pacientes submetidos a esse método nutricional^(18)^. Em um estudo tcheco foi demonstrado que a fistuloclise maior melhora da função hepática que a nutrição enteral, levando a otimização mais rápida dos parâmetros nutricionais, diminuindo o tempo de internação e reduzindo a mortalidade geral dos casos^(19)^.

Existem vários métodos de implementação, dentre os quais podemos destacar o utilizado neste estudo. Os métodos variam desde a aplicação por gravidade até sistemas fechados sofisticados, com a possibilidade de aplicações contínuas^(20,21)^.

O risco mais comum do procedimento é o deslocamento do cateter, que pode ocorrer para o exterior devido a manejo incorreto ou obstrução, ou a interiorização devido ao peristaltismo intestinal^(19)^.

A fistuloclise apresenta sucesso quando a equipe, cuidadores e/ou familiares são envolvidos no processo^(22)^. Neste estudo, a capacitação à beira do leito, conduzida entre a equipe de enfermagem e os familiares, enfrentou resistência, a princípio devido à falta de compreensão sobre a importância do procedimento. No entanto, essa resistência foi diminuída após o treinamento e explicações detalhadas sobre a importância do método. Para quem não conseguiu participar do treinamento presencial, foi elaborado um vídeo instrucional, com o passo a passo do procedimento. A fim de garantir uma alta segura, os cuidados pelos familiares durante os últimos dias de internação foram acompanhados para sanar qualquer tipo dúvida.

O manejo da fistuloclise apresentou barreiras significativas por parte dos enfermeiros estomaterapeutas e dos pacientes, com a recusa em administrar o conteúdo entérico que foi removido do próprio organismo, além de preocupações estéticas por parte dos pacientes e familiares. A educação constante e adequada pela equipe multiprofissional pode diminuir essas barreiras^(1,2,23)^.

A colaboração entre o paciente, família e a equipe facilitará uma abordagem abrangente e eficaz do cuidado. Cada membro da equipe deve realizar avaliações específicas de suas especialidades e colaborar com outros membros da equipe antes de estabelecer metas mutuamente acordadas. Os objetivos devem incluir a manutenção do conforto e da dignidade, atendendo às necessidades psicossociais do paciente com promoção da sua independência^(24)^.

Destaca-se o trabalho do enfermeiro especialista estomaterapeuta que desempenha um papel fundamental na assistência à saúde, especialmente no cuidado de pessoas com estomas e FEA. Esse profissional é altamente especializado e treinado para oferecer um suporte técnico e emocional para melhorar a qualidade de vida dos pacientes^(25)^.

Como implicações e potencialidades para a prática, é possível afirmar que a fistuloclise proporciona resposta nutricional que melhora a condição clínica e recuperação do paciente. A melhora do quadro proporcionada pelo procedimento interfere positivamente na qualidade de vida e acelera a alta hospitalar. No domicílio é possível o manejo da fistuloclise, desde que os familiares sejam capacitados e acompanhados.

As limitações devem ser consideradas como a falta de protocolos pré-estabelecidos para a realização e o cuidado da fistuloclise. As referências utilizadas são publicações de outros países que podem trazer diferenças devido ao acesso a tecnologias e outros recursos não disponíveis no Brasil. Outra limitação é o fato de ser caso único para ser descrito no estudo, mas refere-se à ausência de demais pacientes que realizaram o mesmo procedimento. Importante destacar ainda a dificuldade e resistência encontradas tanto nos profissionais de saúde quanto na família para a realização do procedimento.

A descrição da técnica de fistuloclise associada a discussões sobre o caso pode proporcionar subsídios para que outros profissionais no Brasil desenvolvam o procedimento e publiquem novos casos em periódicos e eventos científicos.

## CONCLUSÃO

A descrição da técnica da fistuloclise da FEA permitiu sua reprodução de forma segura para o controle de efluentes, restauração nutricional e demais respostas clínicas. Destacam-se os cuidados prestados pelo enfermeiro estomaterapeuta, levando em conta os benefícios, a complexidade e os desafios da fistuloclise.
